# Predictors of sustained return to work after sick leave due to mental disorders

**DOI:** 10.47626/1679-4435-2025-1360

**Published:** 2025-07-08

**Authors:** Bruna Roberta Muntanelli, João Silvestre Silva-Junior

**Affiliations:** 1 Departamento de Medicina Legal, Bioética, Medicina do Trabalho e Medicina Física e Reabilitação, Faculdade de Medicina, Universidade de São Paulo, São Paulo, SP, Brazil

**Keywords:** return to work, review, mental disorders., retorno ao trabalho, revisão, transtornos mentais.

## Abstract

Mental disorders are brain dysfunctions that impair occupational performance and are associated with prolonged absences from work and a higher likelihood of relapse. Sustained return to work refers to resuming either original or modified job duties for at least 1 month without recurrent sick leave. This outcome is linked to improved health indicators among workers and reduced socioeconomic burdens resulting from prolonged absences. This study aimed to identify predictors of sustained return to work following sick leave due to mental disorders, based on a literature review conducted in the MEDLINE/PubMed, Scopus, and SciELO databases, including articles published up to July 2023. Quantitative studies were included if they clearly defined sustained return to work and involved workers on leave due to mental disorders. Predictors were categorized into sociodemographic, clinical, psychosocial, and occupational domains. Positive predictors of sustained return to work included favorable expectations regarding the sick leave, self-efficacy, supervisor support, notification systems for prolonged absences, and partial sick leave arrangements. Negative predictors included a greater number of mental disorder episodes, older age, persistent symptoms, job strain, psychiatric/psychological follow-up, medication use, extended duration of leave, employment in small enterprises or specific sectors, and higher educational attainment. Female sex emerged as an inconsistent predictor, with mixed findings. In conclusion, sustained return to work is influenced by multiple factors and results from the interaction between individual and contextual elements. Therefore, collaboration among all stakeholders is essential for successful worker reintegration.

## Abstract (pt)

O transtorno mental é uma disfunção cerebral que prejudica o desempenho profissional, sendo associado a longos períodos de afastamento e maior probabilidade de recaída. O retorno sustentado ao trabalho é o retorno às atividades laborais, originais ou modificadas, por um mínimo de 1 mês, sem novos afastamentos, estando associado à melhora dos desfechos em saúde do trabalhador e à mitigação dos impactos socioeconômicos decorrentes da ausência. Este estudo visou identificar os preditores do retorno sustentado ao trabalho após afastamento por transtornos mentais, por meio de revisão bibliográfica nas bases de dados MEDLINE/PubMed, Scopus e SciELO, com artigos publicados até julho de 2023. Foram incluídos estudos quantitativos que definiram claramente o retorno sustentado ao trabalho e envolveram trabalhadores afastados por transtornos mentais. Os preditores foram classificados nas dimensões sociodemográficas, clínicas, psicossociais e ocupacionais. Preditores positivos para o retorno sustentado ao trabalho incluíram expectativas favoráveis sobre o afastamento, autoeficácia, suporte do supervisor, notificações sobre afastamentos prolongados e afastamentos parciais. Preditores negativos foram maior número de episódios de transtornos mentais, idade avançada, persistência de sintomas, pressão no trabalho, acompanhamento psiquiátrico/psicológico, uso de medicação, tempo prolongado de afastamento, vínculo com pequenas empresas e atuação em certos setores, além de alta escolaridade. O sexo feminino foi um preditor dúbio, com resultados inconsistentes. Conclui-se que o retorno sustentado ao trabalho é multifatorial, dependendo da interação entre fatores individuais e contextuais, exigindo colaboração entre os envolvidos na reintegração do trabalhador.

## INTRODUCTION

Mental disorders are conditions that affect cognition, emotion, and behavioral control, leading to significant impairment in personal and professional functioning, as well as in social relationships.^1^ In 2019, one in eight people worldwide were diagnosed with anxiety disorders or major depression, resulting in an estimated loss of 12 billion workdays due to disability and a global productivity loss of US$1 trillion.^2^ In Brazil, the National Institute of Social Security (Instituto Nacional do Seguro Social [INSS]) is responsible for providing benefits to insured individuals who are temporarily unable to perform their work activities due to illness for a period exceeding 15 days. According to data published in the Social Security Statistical Yearbook,^3^ mental health conditions were the third leading cause of temporary work disability in 2021, accounting for 9% of all granted social security benefits and 10% of the total benefit-related costs.

Mental disorders are associated with longer periods of work absence compared to other health conditions,^4^ and the likelihood of returning to work decreases progressively with the length of time away from work.^5^ Recurrence of disabling mental disorders is common and may affect 20% to 30% of workers who return to work within a 10-year period.^6^ These relapses are typically more severe and result in longer absences than the initial episode.^7^ Prolonged work absences place a financial burden on employers, increase social security expenditures, and hinder the worker’s reintegration into the workplace and society.^8^

Returning to work is the next step after the end of a sick leave period and represents a critical phase in the process of reintegrating the worker into their professional activities. Understanding the factors that promote continued work participation and reduce the likelihood of relapses is essential. In the literature, there is no consensus on the definition of sustained return to work (SRTW). In most studies,^6,9,10^ SRTW is defined as a period of 4 consecutive weeks without a new episode of sick leave. This duration is considered sufficient to suggest a stable level of work functioning. Corbière et al.^11^ propose that the sustainability of return to work should be assessed 3 months after resumption. Arends et al.^12^ used follow-up assessments at 3, 6, and 12 months post-return. Lammerts et al.^13^ suggest that the outcome should be evaluated after 2 years of work stability.

Some studies require that the worker return to the same workload as before the leave,^14,15^ while others consider partial return to work to be acceptable.^11,16^

Despite variations in its definition, SRTW is understood to result from a complex interplay of individual, behavioral, and environmental factors.^17,18^ The worker’s interaction with the workplace can play a crucial role in rehabilitation and reintegration.^17,19^ Previous studies have shown that supervisor support and effective communication among colleagues are essential for SRTW, particularly in cases involving mental disorders.^20^ SRTW is associated with better health outcomes for workers, reduced risk of recurrent leave or permanent disability, and minimized social and economic consequences of absence.^8^

Although the impacts of work absence due to mental disorders are well recognized, and the importance of SRTW is acknowledged, the predictive factors associated with successful reintegration remain poorly understood. Identifying these factors may help guide strategies aimed at ensuring a lasting return to work. The objective of this review is to identify the main sociodemographic, clinical, psychosocial, and occupational predictors of SRTW following sick leave due to mental disorders.

## METHODS

A literature review was conducted using original articles published up to July 31, 2023, in the MEDLINE/PubMed, Scopus, and SciELO databases. The search strategy combined keywords and Boolean operators as follows: (((sustainable OR sustained) AND return to work) AND mental).

Inclusion criteria were as follows: participants had to be workers on sick leave due to a mental disorder; SRTW had to be clearly defined as no new episodes of sick leave within a period shorter than one month (28 or 30 days) after returning to work, including return to regular or modified work activities, either full-time or part-time. Exclusion criteria included: review articles, qualitative studies, and studies whose outcomes did not align with the objective of this review..

No restrictions were applied regarding the time frame of article publication, nor were there limitations related to participants’ sex, age, nationality, or sociocultural characteristics. All relevant studies published prior to the search date were included. Study selection followed the Preferred Reporting Items for Systematic Reviews and Meta-Analyses (PRISMA) flowchart model.^21^

From the selected articles, quantitative statistical inference results were extracted, specifically those relating the outcome to explanatory variables. General information from the selected studies is presented in Table 1, including the following details: author, year of publication, country of origin, study design, follow-up duration, sample size, mean age, and sex distribution. Subsequently, key information from each included study was compiled and organized to include author, study population, outcome of interest, definition and incidence of SRTW, and the identified positive, negative, or inconclusive predictors of SRTW, as shown in Table 2. Predictors were classified as positive, when RTW was achieved; negative, when it was unsuccessful; and unclear, when the findings were inconsistent.

**Table 1:** Overall characteristics of the studies included in the review of sustained return to work following leave due to mental health disorders

Author	Year	Country	Study design	Follow-up duration	Participants (n)	Age (mean)	Sex (%) (M/F)
Mishima et al.^22^	2020	Japan	Prospective cohort	3 years	234	37.0 (O)	91.5/8.5
Aasdahl et al.^18^	2019	Norway	Prospective cohort	9 months	168	47.0 (O)	19.0/81.0
Black et al.^23^	2019	Australia	Prospective cohort	12 months	410	45.1 (O)	44.3/55.7
Jetha et al.^24^	2018	Australia	Prospective cohort	6 months	551	-	48.2/51.9
Halonen et al.^25^	2018	Finland	Retrospective cohort	2 months	50.426 (cohort 1) 52.789 (cohort 2)	-	42.7/57.3 (cohort 1) 41.9/58.1 (cohort 2)
Kausto et al.^26^	2017	Finland	Prospective cohort	6 years	123.506	43.1 (M)/42.7 (F)	24.3/75.7
Viikari-Juntura et al.^10^	2017	Finland	Prospective cohort	2 years	3.756 (case) 1.878 (control)	Caso: 44.6 (M)/45.6 (F) Controle: 45.9 (M)/46.9 (F)	22.9/77.1 (case) 22.3/77.2 (control)
Prang et al.^27^	2016	Australia	Retrospective cohort	2 years	8.358	44.0 (O)	44.0/56.0

F = female; O = overall; M = male.

**Table 2:** Key information from each study included in the review: author, population, outcome of interest, definition and incidence of SRTW, and positive and negative predictors of following leave due to mental disorders

Author	Population	Assessed outcome	SRTW		Predictors	
			Definition	Incidence	Positive	Negative
Mishima et al.^22^	Automotive industry workers in Japan on sick leave for at least 30 days due to mental disorders	Relationship between the number of prior mental disorder episodes and SRTW	Return to work with no new sick leave episodes for at least 30 days	23-86% over 36 months	-	Higher number of previous mental disorder episodes
Aasdahl et al.^28^	Workers on leave for 2-12 months due to mental, musculoskeletal, or general/unspecified disorders	Expectations about leave duration and SRTW	1 month without receiving sickness benefits after returning to work	41% over 9 months	Positive expectations about duration of leave	-
Black et al.^23^	Workers on leave for 4-6 months due to mental or musculoskeletal disorders	Relationship between return-to-work self-efficacy and SRTW	Return to regular or modified duties with no new sick leave for at least 28 days	37.9% at 6 months; 21.5% at 12 months	Return-to-work self-efficacy during early recovery	
Jetha et al. ^24^	Workers on leave, recruited on average 3-4 months after work-related psychological/mental or musculoskeletal injuries	Social impact of workplace environment and SRTW	Return to regular or modified duties with no new sick leave for at least 28 days	59% at baseline; 70% after 6 months	Positive supervisor response to the ill worker	-
Halonen et al.^25^	Workers on leave for >30 days due to various disorders (musculoskeletal, mental, injury, cardiovascular, neurological, cancer, others)	Impact of official mandatory notification of extended absences (>30 days) and SRTW	Return to full-time work for at least 28 consecutive days	-	Official notification of extended absences (>30 days)	-
Kausto et al.^26^	Workers with musculoskeletal, mental health, injury, cardiovascular, neurological, cancer, and other disorders as well as subsequent SWTR	Association between duration of sick leave and SRTW and its predictors	Return to work with no new sick leave episodes for at least 30 days	80% (depression); 93% (anxiety disorders)	Female sex (in cases of major depression)	Older age, persistent health problems in both sexes, and comorbidities in women
Viikari-Juntura et al.^10^	Workers on full or partial leave due to mental or musculoskeletal disorders	Effectiveness of partial sick leave and SRTW	Return to regular work duties, fullor part-time, for at least 28 consecutive days	77.5%	Partial sick leave	-
Prang et al.^27^	Workers on leave for at least 10 days due to mental disorders (stress-related disorders, posttraumatic stress disorder, or other mental illness)	Predictors of SRTW in workers on leave due to mental disorders	Full return to pre-injury work with no new absences or benefits for at least 30 days	93.9% over 2 years	-	Female sex, older age, bullying/harassment or pressure as mechanism of illness onset, psychiatric/psychological follow-up, prescribed medication use, higher number of prior episodes, longer leave duration, employment in small enterprises or specific sectors (manufacturing, trade, telecommunications, public services, education, finance), high skill level (education)

SRTW = sustained return to work.

Finally, the identified predictors were categorized according to different domains, including sociodemographic, clinical, psychosocial, and occupational aspects, as detailed in Table 3. Predictors were considered statistically significant when the p-value was less than 0.05 with a 95% CI.

**Table 3:** Statistically significant predictors of sustained return to work categorized by domain and outcome

Predictors	Positive	Negative	Inconclusive
Sociodemographic		High qualification (high educational level or >5 years of training) (1)	Sex (2)
Clinical		Number of episodes (2) Comorbidities (1) Psychiatric/psychological follow-up (1) Medication use (1) Depression (1) Persistence of health problems (1)	
Psychosocial	Positive expectation of returning to work (1) Self-efficacy (1)		
Occupational	Supervisor support (1) Partial sick leave (1) Notification of prolonged absences (1)	Duration of sick leave (1) Bullying/harassment or pressure at work as a mechanism of illness onset (1) Employment in small enterprises (1) Employment in the following sectors: manufacturing, commerce, telecommunications, public administration or security, education, financial sector (1)	

## RESULTS

**Figure 1:**
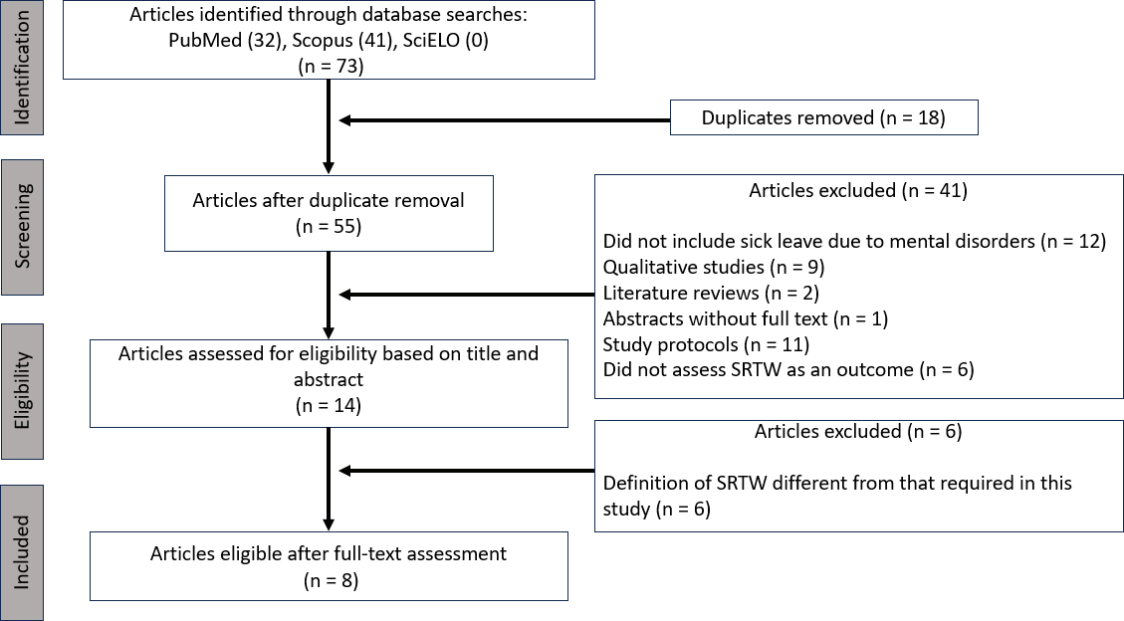
Article selection process according to the Preferred Reporting Items for Systematic Reviews and Meta-Analyses (PRISMA) model. SRTW = sustained return to work.

A total of 73 studies were identified in the databases, of which 18 were excluded due to duplication. After screening titles and abstracts, 41 studies were excluded for the following reasons: they did not include workers on leave due to mental disorders (n = 12), were literature reviews (n = 2), qualitative studies (n = 9), study protocols (n = 11), abstracts without full-text availability (n = 1), or did not assess SRTW as an outcome (n = 6). Of the 14 studies eligible for full-text review, 6 were excluded because they used a definition of SRTW that did not meet this study’s criteria. After full-text assessment, eight studies were included in this review. Figure 1 presents the article selection process according to the PRISMA model.

All eight selected studies were conducted in socioeconomically developed countries - three in Finland, three in Australia, one in Norway, and one in Japan. All included studies employed a cohort design: six were prospective and two retrospective, with follow-up durations ranging from 2 months to 6 years. Sample sizes varied from 168 to 123,506 participants, with the largest populations found in retrospective cohorts. Seven of the eight studies included workers whose sick leave data were obtained from national social security systems. One study included only public sector workers. Another study focused exclusively on workers from the automotive industry, with leave-related data obtained from company records or patient medical files.

All studies included both male and female workers in varying proportions. In three studies, the proportion of female participants exceeded 75% (75.7-81.0). In one study, 91.5% of participants were male. Only one study included workers who were on leave exclusively due to mental disorders.

## DISCUSSION

Mishima et al.^22^ investigated whether the number of previous episodes of work absence due to mental disorders was associated with SRTW. The study consisted of a cohort of 234 workers from a Japanese automotive industry who had been on sick leave for at least 30 days due to a mental disorder. Eligibility criteria included being between 18 and 60 years old, currently employed, and having a mental disorder diagnosed by a psychiatrist. Participants were divided into three groups based on the number of previous episodes of mental disorders: one episode (group 1), two episodes (group 2), and three or more episodes (group 3), and were followed for 3 years. The outcome assessed was the SRTW rate in each group at 6, 12, 18, 24, 30, and 36 months. SRTW was defined as returning to work without new sick leave episodes for at least 30 consecutive days. The study found that the SRTW rate was significantly higher in group 1 compared to group 3 across all time points. This result suggests that repeated episodes of work absence due to mental disorders are predictive of a poorer prognosis for sustained return to work.

Aasdahl et al.^18^ conducted a prospective cohort study with a 9-month follow-up involving 168 workers randomized into two rehabilitation therapy protocols (Acceptance and Commitment Therapy [ACT]). Eligible participants were workers aged 18-60 who had been on at least 50% sick leave for a period of 2 to 12 months due to mental or musculoskeletal disorders. Sick leave data were obtained from the Norwegian national social security system. The aim of the study was to assess whether participants’ expectations regarding the duration of their sick leave changed during occupational rehabilitation, and whether this change was associated with SRTW. Data were collected using questionnaires administered at the beginning and end of the rehabilitation program. SRTW was defined as working for at least one month without receiving medical benefits. Data analysis was adjusted for age, gender, educational level, rehabilitation program, duration of sick leave, self-rated health, and employment status. SRTW was achieved by 41% of participants. The study found that participants who consistently held positive expectations regarding the duration of their sick leave had a higher likelihood of achieving SRTW, suggesting that such expectations are a strong predictor of sustained return to work.

Black et al.^23^ conducted a prospective cohort study with a 12-month follow-up involving 410 workers on leave due to mental or musculoskeletal disorders. Sick leave data were obtained from the occupational health and safety regulatory system of the state of Victoria, Australia (WorkSafe Victoria). Eligible participants were workers who had sustained a mental or musculoskeletal injury and were on leave either at T1 (4-6 months post-injury) or T2 (10-12 months post-injury). The objective of the study was to evaluate the relationship between return-to-work self-efficacy and SRTW, and how this relationship changed over the 12-month period. Data were collected through questionnaires administered at three time points: 4-6 months (T1), 10-12 months (T2), and 16-18 months (T3) post-injury. The outcome assessed was SRTW at 6 and 12 months after T1. SRTW was defined as a return to the same type of work as before the leave or to modified/adapted duties for at least 28 consecutive days. Data analysis was adjusted for age, gender, type of injury, illness duration, prior employment status, job autonomy, and interaction with the return-to-work coordinator. Return-to-work self-efficacy was positively associated with SRTW at T2 (10-12 months post-injury), but not at T3 (16-18 months post-injury). These findings suggest self-efficacy is a positive predictor of sustained return to work during the early stages of recovery.

Jetha et al.^24^ conducted a 6-month prospective cohort study involving 551 workers aged 18 and older who were on leave due to work-related psychological/mental or musculoskeletal conditions. Sick leave data were obtained from the occupational health and safety regulatory system of the state of Victoria, Australia (WorkSafe Victoria). The objective of the study was to examine the social impact of the work environment on SRTW. The sample of workers on leave was recruited over a 1-year period, as soon as their benefit claims were approved. After recruitment, participants were interviewed at baseline (Time 1) and contacted again 6 months later for a follow-up interview (Time 2). Social support and reactions to the injury were assessed at both time points, across two dimensions: (1) social support from coworkers and supervisors, and (2) coworkers’ and supervisors’ reactions to the worker’s illness. SRTW was assessed at both time points and was defined as remaining at work, with no new sick leave, for at least 28 days following return - whether to the same role or to a modified one. At Time 1, 59% of participants reported SRTW, and at Time 2, 70% did. The final analysis showed that a positive supervisor reaction to an ill worker was associated with SRTW at both time points, highlighting the critical role of the supervisor in facilitating a sustained return to work.

Halonen et al.^25^ investigated whether the proportion of workers who achieved SRTW changed following a 2012 legislative reform in Finland, which mandated that employers notify occupational health services of prolonged sick leave episodes (>30 days). The study included two cohorts, each comprising approximately 50,000 workers, with a follow-up period of 2 months. Seventy percent of working-age individuals (18-60 years) residing in Finland in 2010 (pre-intervention) and 2013 (post-intervention) were included. Participants had been on sick leave for more than 30 days due to various health conditions, 13.9% of which were mental disorders. The outcome assessed was the average time to SRTW at the end of the 2-month follow-up. SRTW was defined as returning to full-time work for at least 28 consecutive days. The analysis was stratified by sex, age group, employment sector (public/private), diagnosis (musculoskeletal, mental, acute injury, cardiovascular, neurological, cancer, other), and unemployment rate (high/low). The results indicated that SRTW was 5% higher in the post-intervention period for workers on leave due to mental disorders.

Kausto et al.^26^ conducted a cohort study involving 123,506 public sector workers in Finland, followed over a 6-year period. The eligible population consisted of employees working in 10 municipalities and six hospital districts. The objective was to investigate the association between the duration of sick leave and SRTW as well as the predictors of SRTW in cases of depression, anxiety disorders, and musculoskeletal disorders among workers who developed these conditions during the study period. SRTW was defined as the end of a sick leave period not followed by a new episode of sick leave within 30 days. The main variables analyzed were age, sex, occupational group, region of the country, persistence of health problems, comorbidities, use of antidepressants, and hospitalization for mental disorders. Among workers on leave due to depression, 80% achieved SRTW. For anxiety disorders, the rate was 93%. In cases of depression, the average time to SRTW ranged from 33 to 57 days in men and from 31 to 40 days in women, depending on the age group. Older age (> 46 years) was associated with longer time to SRTW. Persistence of health problems in both sexes and the presence of comorbidities in women were also associated with delays in SRTW. For anxiety disorders, the average time to SRTW ranged from 22 to 27 days in men, depending on age. In men, older age and persistence of health problems were associated with longer time to SRTW. In women, persistence of health problems was the key associated factor.

Viikari-Juntura et al.^10^ conducted a cohort study involving 3,756 workers aged 20 to 64 who were on sick leave due to mental or musculoskeletal disorders. Eligibility criteria included being on partial (case group) or full-time (control group) sick leave. Since 2007, Finnish legislation has allowed workers to return to their jobs on a partial basis - working 40% to 60% of their regular hours - after being fully absent for 60 days due to illness. The aim of the study was to evaluate the effectiveness of partial sick leave during the early stages of work disability in achieving SRTW. The primary outcome was SRTW, defined as returning to regular work duties, either full-time or part-time, for at least 28 consecutive days. The results showed that 77.5% of the study population achieved SRTW during follow-up, with a higher proportion of SRTW observed among workers who returned on a partial basis. This difference was consistent across both sexes, all age groups, and among cases involving mental disorders.

Prang et al.^27^ conducted a retrospective cohort study with a two-year follow-up involving 8,358 workers who had been on sick leave for at least 10 days due to a mental disorder. Eligible participants were between 15 and 70 years of age. The primary outcome of interest was the time to first SRTW over the two-year follow-up period. SRTW was defined as a full return to the same work duties as before the absence, with no new episodes of sick leave or benefit claims for at least 30 consecutive days. A total of 93.9% of workers achieved SRTW. Among those who did not, the average age was 47 years, 57.2% were women, 85.4% had a stress-related mental disorder, 30.4% reported bullying or harassment, and 27% identified workplace pressure as a contributing factor to their condition. The study found evidence suggesting that women, older individuals, those employed by small businesses or in certain sectors (manufacturing, trade, telecommunications, public administration or security, education, and finance), workers in occupations requiring high qualifications (advanced education or > 5 years of training), those undergoing psychiatric or psychological follow-up, individuals using prescribed medications, those with a history of prior episodes, and those with longer leave durations were more likely to face difficulties in achieving SRTW.

Scientific data on SRTW following sick leave due to mental disorders remain scarce. Although no time restriction was applied during the literature search, the earliest study identified on this topic was published in 2014. This suggests that the subject has only recently gained relevance in the global scientific landscape, largely due to the rising number of sick leaves caused by mental disorders, their socioeconomic consequences, and future projections by the World Health Organization (WHO), which indicate that by 2030, mental health conditions will be the leading cause of disability worldwide.^29^

In two studies,^22,27^ the number of previous episodes of mental illness was identified as a strong negative predictor of SRTW. Mishima et al.^22^ reported that the incidence of SRTW after one prior episode was 86% at 6 months and 57% at 36 months. In contrast, among participants with three or more prior episodes, these rates were 63% and 26%, respectively, over the same periods. These findings suggest that repeated absences due to mental disorders indicate a poorer prognosis for SRTW, with a progressively increasing likelihood of relapse over the three years following return to work. In a previous study, Sado et al.^30^ also found that the number of sick leave episodes due to mental disorders was a significant predictor of future leave, although it did not specifically assess the relationship with SRTW. Given the potential association between the number of episodes and SRTW, early diagnosis and treatment of mental disorders, along with proper follow-up during the return-to-work process, should be prioritized to help prevent relapse.

According to Aasdahl et al.,^18^ holding positive expectations regarding the duration of sick leave is a relevant positive predictor of achieving SRTW. This finding is supported by the results of Black et al.^23^, which indicate that self-efficacy positively predicts SRTW in workers. Self-efficacy refers to an individual’s belief in their own ability to succeed in a given task.^31^ The association between return-to-work expectations and work-related outcomes appears to be a stronger predictor than clinical variables.^32^ Developing strategies that help workers strengthen their sense of self-efficacy may serve as an important tool for coping with challenges, contributing to greater motivation and more positive expectations in the face of adversity.

Jetha et al.^24^ aimed to evaluate the impact of the workplace social environment on SRTW. Their results suggest that a positive reaction from the supervisor toward the ill worker is an important predictor of SRTW. In the same study, coworker support and their reaction to the worker’s injury were not significantly associated with SRTW. Previous research^33^ has already indicated that the work environment - particularly the way supervisors interact with employees - can either facilitate or hinder the return-to-work process. Supervisor support may contribute to an earlier, more sustained return. Similarly, Prang et al.^27^ found that a hostile work environment characterized by bullying, harassment, or high pressure negatively impacts SRTW. These findings underscore the need for organizational policies that promote effective communication between workers and supervisors as a strategy to facilitate reintegration and support sustained return to work.

Older age emerged as a negative predictor of achieving SRTW in two studies.^26,27^ Older individuals are more likely to experience chronicity of illness^34^ and to present with comorbid conditions associated with mental disorders. The persistence of health problems has been shown to predict longer time to SRTW in cases of depression, in both men and women.^26^

Prang et al.^27^ also identified other significant negative predictors of SRTW: female sex, psychiatric or psychological follow-up, use of prescribed medication, longer duration of sick leave, employment in small companies, and work in certain sectors, as well as higher educational attainment. Conversely, Kausto et al.^26^ found that being female was a positive predictor of SRTW in cases of depression, with women presenting a shorter time to SRTW. Psychiatric follow-up or use of medication may indicate greater severity of illness, which can result in greater difficulty achieving SRTW. The same is true for cases requiring longer periods of leave. Occupations that require high levels of qualification are often associated with increased stress due to greater responsibilities and demands, which may pose additional challenges to the SRTW process.

All studies were conducted in developed countries, where social and health policies targeting workers - particularly those focused on mental health and well-being - are more commonly implemented. One study^25^ evaluated the impact of a 2012 legislative reform in Finland requiring employers to notify the national Occupational Health and Safety (OHS) system when employees are on sick leave for more than 30 days. The findings indicated an increase in SRTW rates and a reduction in time to SRTW following the implementation of the legislation, with more pronounced effects observed among women and workers on leave due to mental disorders. The significance of this study lies in demonstrating that the creation of public policies aimed at enhancing communication between employers, workers, and national regulatory bodies can serve as an important strategy for managing long-term sick leave due to mental disorders. These cases often require careful planning and coordination throughout the return-to-work process.

Along similar lines, Viikari-Juntura et al.^10^ investigated whether the legislation introduced in Finland in 2007 - which allows workers to return to work on a part-time basis (40-60% of their previous working hours) after sick leave - affected SRTW rates compared to full-time return. A higher proportion of SRTW was observed among those who returned on a part-time basis, with the difference being particularly significant for leaves due to mental disorders. This finding was consistent across all age groups, employment sectors (public vs private), and occupational categories.

In Brazil, although significant progress has been made through the Consolidation of Labor Laws (CLT, 1943), which regulates workers’ rights, and the establishment of the INSS, responsible for meeting the social and welfare needs of workers, the country still lacks a structured return-to-work program. Regulatory Standard No. 7 (NR-7), established in 1978, governs the Occupational Health Medical Control Program (Programa de Controle Médico de Saúde Ocupacional [PCMSO]), whose primary goal is to promote and preserve workers’ health. NR-7 states that, during the return-to-work medical examination, the physician must assess the need for a gradual reintegration into work activities.

Despite these provisions, no formal program exists in Brazil to operationalize a gradual return-to-work process, which hinders its implementation within companies. In Norway, for example, the Norwegian Labour and Welfare Administration (NAV) plays a central role in monitoring workers on sick leave and coordinating their return to work. NAV facilitates collaboration and dialogue involving the ill worker, the employer, and the physician.^28^ In Finland, in addition to the option of part-time return, the social security system provides wage supplementation for workers returning on a reduced schedule, which serves as an incentive for companies to support the reintegration process.

This complex return-to-work process should not fall solely under the responsibility of the occupational physician conducting the return-to-work evaluation. It must involve active participation from employers, leadership, and government entities, as it encompasses financial, organizational, and systemic issues that extend beyond the scope of occupational health professionals.

In 2019, Etuknwa et al.^19^ conducted a systematic review aimed at identifying personal and social factors that predict SRTW. According to the study’s findings, the most consistent predictors of SRTW in cases of mental disorders included supervisor and coworker support, positive attitude and self-efficacy, younger age, and higher educational attainment. Duration of sick leave also showed promising associations, while findings related to gender were inconclusive. These results are relatively consistent with the findings of the present review.

A major strength of the present study lies in its focus on a relevant and timely topic, considering the growing impact of mental disorders in the workplace and the need for effective strategies to support sustained return to work. The review enabled the identification and categorization of key SRTW predictors, providing a broad overview of the subject and supporting future research. Additionally, the inclusion of studies from various countries and contexts allowed for comparisons across different realities and reinforced the importance of institutional policies in promoting SRTW.

However, some limitations should be acknowledged. The review was based exclusively on studies conducted in developed countries, where public policies and social security systems differ significantly from the Brazilian context, limiting the generalizability of findings to the national setting. Furthermore, the scarcity of Brazilian research on the topic hinders the development of context-specific guidelines and highlights the need for future studies that address the challenges and opportunities related to SRTW within the local context. Another limitation concerns the methodological approach, as this narrative review did not follow a systematic process for study selection and analysis, potentially introducing selection and interpretation bias. In addition, the lack of quantitative criteria makes it difficult to assess the quality of the evidence, reducing the generalizability of the findings.

## CONCLUSIONS

This review identified and categorized the main predictors of SRTW following sick leave due to mental disorders, highlighting the complexity of the process and the interaction among sociodemographic, clinical, psychosocial, and occupational factors. Self-efficacy and organizational support - particularly from supervisors - emerged as positive predictors, while multiple previous episodes of leave, prolonged periods of inactivity, older age, and hostile work environments were associated with difficulties in achieving SRTW. Institutional strategies, such as partial sick leave and occupational rehabilitation programs, demonstrated potential to improve return-to-work rates and their long-term sustainability.

Despite advances in understanding these factors, the literature on the topic remains limited, particularly in Brazil, where studies evaluating effective interventions to ensure a sustainable return to work are lacking. All studies included in this review were conducted in developed countries, whose public policies and social security systems differ significantly from the Brazilian context. This highlights the need for research that considers the specific characteristics of the Brazilian labor market.

The findings reinforce the importance of integrated approaches to managing SRTW, involving not only clinical follow-up of the worker but also adaptation of working conditions and strengthening of organizational support. Implementing policies that facilitate communication among employers, health care services, and social security systems may help reduce the recurrence of sick leave and promote sustained reintegration of workers. As mental disorders increasingly affect the workplace and carry significant economic and social costs, more effective strategies are urgently needed to support a safe, productive, and sustained return to work.
